# An animal-type Na^+^/K^+^-ATPase, *PhNKA2*, is involved in the salt tolerance of the intertidal macroalga *Pyropia haitanensis*

**DOI:** 10.3389/fpls.2025.1571241

**Published:** 2025-04-28

**Authors:** Rongrong Feng, Qi Chen, Yan Xu, Dehua Ji, Chaotian Xie, Wenlei Wang

**Affiliations:** ^1^ Fisheries College, Jimei University, Xiamen, China; ^2^ State Key Laboratory of Mariculture Breeding (Jimei University), Ningde, China; ^3^ Key Laboratory of Healthy Mariculture for the East China Sea, Ministry of Agriculture and Rural Affairs, Xiamen, China

**Keywords:** salt stress, intertidal seaweed, animal-type Na^+^/K^+^-ATPase, heterologous expression, yeast two-hybrid

## Abstract

Intertidal red algae, are more tolerant to salt stress than terrestrial plants, contain a Na^+^ transporter (Na^+^/K^+^-ATPase) that is homologous to animal Na^+^/K^+^-ATPases. Although two Na^+^/K^+^ pump genes from Pyropia/Porphyra were cloned and their differential expression patterns under salt stress were analyzed, the regulatory mechanism of Na^+^/K^+^-ATPase genes in Na^+^ expulsion and K^+^ retention process under salt stress remains largely unknown. In this study, we cloned and characterized the animal-type Na^+^/K^+^-ATPase gene *PhNKA2* in *Pyropia haitanensis*. The encoded protein was revealed to contain an N-terminal cation-transporting ATPase, E1/E2 ATPase, hydrolase, and a C-terminal cation-transporting ATPase. *PhNKA2* was highly conserved in *Porphyra*/*Pyropia*. The expression of *PhNKA2* in gametophytes was significantly induced by hypersalinity, while there was no obvious change in sporophytes. The heterologous expression of *PhNKA2* in *Chlamydomonas reinhardtii* clearly increased salt tolerance. Na^+^ efflux and K^+^ influx were significantly greater in the transgenic *C. reinhardtii* than in the wild-type control. Furthermore, yeast two-hybrid assays suggested that the interaction between the deubiquitinating enzyme USP5 and *PhNKA2* might be critical for the deubiquitination and stabilization of important proteins during the *P. haitanensis* response to salt stress. The interaction with MSRB2, DHPS, or GDCST may prevent the oxidation of *PhNKA2*, while actin depolymerization might stimulate Na^+^/K^+^-ATPase-dependent membrane trafficking. The results of this study provide new insights into the salt tolerance of intertidal seaweed as well as the underlying molecular basis.

## 1 Introduction

In plants, the most adverse effect of salt stress is the ion toxicity due to high Na^+^ concentrations. The accumulation of Na^+^ inhibits plant growth, development, and energy metabolism, leading to premature aging and even death (Yang and Guo, 2018). Additionally, because Na^+^ and K^+^ have similar properties, Na^+^ partially replaces K^+^ and restricts the influx of K^+^. An excessive amount of Na^+^ can result in the depolarization of the cell membrane, leading to the efflux of K^+^ and an ionic imbalance within the cell, which alters normal plant growth. Therefore, maintaining the K^+^/Na^+^ balance is an essential part of the plant adaptive response to salt stress (Zhu, 2016; Hussain et al., 2021).

Intertidal red algae, including edible seaweed, can maintain the K^+^/Na^+^ balance better than terrestrial plants, seagrass, and other salt-tolerant plants (Chen et al., 2019). In addition to possessing the highly conserved SOS1 protein found in plants, red algae also have an animal-like Na^+^-K^+^ pump, which is reportedly absent in flowering plants (Uji et al., 2012; Chen et al., 2022). This difference between red algae and flowering plants may be explained by the fact a low environmental Na^+^ concentration means a system that mediates extensive Na^+^ efflux is unnecessary. Accordingly, Na^+^-K^+^ pumps were likely lost during evolution in fresh water environments (Benito and Rodríguez-Navarro, 2003). Moreover, in low-Na^+^ environments, generating a steep electrochemical gradient that energizes the plasma membrane using Na^+^ is impossible (Pedersen et al., 2010). We previously determined that using ouabain to inhibit the Na^+^-K^+^ pump results in the significant accumulation of Na^+^ and a noticeable decrease in the K^+^ content in intertidal seaweed *Pyropia haitanensis*. The resulting significant decrease in salt tolerance reflects the importance of the Na^+^-K^+^ pump for maintaining the K^+^/Na^+^ balance in intertidal seaweed exposed to salt stress (Chen et al., 2022).

The animal-type Na^+^/K^+^-ATPase, which was the first identified ATPase that functions as an ion transporter, is the most famous and characteristic Na^+^ pump in eukaryotic organisms (Glynn, 1993; Skou, 1998; Kaplan, 2002). Its classical structure consists of α and β subunits that form a binary complex. The α subunit serves as the catalytic subunit with ATP-binding sites involved in ATP catalysis, whereas the β subunit is mainly responsible for stabilizing the conformation of the α subunit and regulating its activity. After Na^+^ enters the cell, it binds to the Na^+^/K^+^-ATPase, resulting in the hydrolysis of ATP and phosphorylation. The associated conformational change in the pump leads to the efflux of Na^+^ from the cell. Simultaneously, extracellular K^+^ binds to the pump, thereby inducing dephosphorylation and restoring the protein conformation, which leads to an influx of K^+^ into the cell and the completion of a full cycle (Scheiner-Bobis, 2002; Morth et al., 2011; Kanai et al., 2021). Each cycle consumes one ATP molecule and pumps out three Na^+^ and pumps in two K^+^ against the electrochemical gradient (Gadsby et al., 2012). Therefore, compared with SOS1, Na^+^/K^+^-ATPase regulates the K^+^/Na^+^ balance more directly and efficiently. Increases in the intracellular Na^+^ concentration and the extracellular K^+^ concentration can activate Na^+^/K^+^-ATPase. The Na^+^/K^+^-ATPase activity allows the cell to maintain the imbalanced distribution of low Na^+^ and high K^+^. Furthermore, the electrochemical Na^+^ gradient generated by Na^+^/K^+^-ATPase serves as the driving force for the secondary transport of various substances across the cell membrane. Therefore, Na^+^/K^+^-ATPase is a key enzyme for maintaining the ionic balance, osmotic balance, and nutrient transport (Takada et al., 2009; Kumari and Rathore, 2020).

The Na^+^/K^+^-ATPase activities have been characterized in various red algal species. Both *Porphyra umbilicalis* and *P. haitanensis* have two genes encoding P2C-type Na^+^/K^+^ ATPases in their genomes (Brawley et al., 2017; Chen et al., 2022). Salt stress significantly induces the expression of *PhNKA2* in *P. haitanensis* thalli, while the transcript level of *PhNKA1* did not increase in thalli under same salinity condition (Chen et al., 2022). Two genes (*PyKPA1* and *PyKPA2*) encoding a homolog of the animal Na^+^/K^+^-ATPase *α* subunit were identified in another economically important red algal species (*Pyropia yezoensis*) via gene cloning (Uji et al., 2012). The *PyKPA1* and *PyKPA2* expression levels are upregulated and downregulated, respectively, in thalli exposed to alkali stress, whereas the expression of both genes in thalli is induced by cold stress. Kishimoto et al. (2013) used transgenic technology to heterologously express the *P. yezoensis* Na^+^/K^+^-ATPase-encoding gene *PyKPA1* in rice. The transgenic rice plants exhibited significantly improved salt tolerance, suggesting that in *Pyropia*/*Porphyra* species, the plasma membrane may be energized with Na^+^ via Na^+^/K^+^-ATPases for secondary active transport, enabling them to survive in the intertidal zone. Although researchers have cloned two Na^+^/K^+^ pump genes from *Pyropia*/*Porphyra*, analyzed their differential expression patterns under salt stress, cold stress, and alkaline stress, and verified their function in enhancing rice salt tolerance through heterologous expression, the regulatory mechanism of Na^+^/K^+^-ATPase genes in Na^+^ expulsion and K^+^ retention process under salt stress remains unclear.

In summary, this study focuses on *PhNKA2* from the economically valuable intertidal seaweed *P. haitanensis*, which is significantly induced by salt stress and various techniques were applied (e.g., molecular cloning, non-destructive micro-measurements, yeast two-hybrid assay) to comprehensively analyze the salt tolerance-related function of the Na^+^/K^+^-ATPase in intertidal seaweed *P. haitanensis*. Additionally, although CRISPR functionality has been confirmed in two brown algae species (*Ectocarpus* sp*ecies 7* and *Saccharina japonica*), two red algae species (*Gracilariopsis lemaneiformis* and *P. yezoensis*), and one green algae species (*Ulva prolifera*), these studies are limited to proof-of-concept demonstrations, and the editing efficiency is relatively low (De Saeger et al., 2024; Wang et al., 2024). Unlike seaweed species, *Chlamydomonas reinhardtii* is a unicellular green alga broadly used for elucidating fundamental biological processes. Many heterologous genes have been expressed in *C. reinhardtii*, including genes from *Pyropia* (Kim et al., 2011; Chang et al., 2021; Wang et al., 2021), higher plants (Siripornadulsil et al., 2002), and humans (Rasala and Mayfield, 2011). Therefore, this study uses *C. reinhardtii* for heterologous expression to verify the biological function of *PhNKA2*. The study findings have clarified the molecular basis of the intertidal seaweed response to salt stress, while also providing new insights relevant to breeding salt-tolerant marine crops.

## 2 Materials and methods

### 2.1 Experimental materials and stress treatments

In this study, *P. haitanensis* strain Z-61 was used. Thallus samples were obtained from the *Pyropia haitanensis* Germplasm Resource Bank in Fujian province, China (Ling-Min, 2008). Thallus samples that were 15 ± 1 cm long and in good condition with a smooth and undamaged surface were selected for the subsequent experiments. The thalli were cultivated at 21 ± 1°C with a light intensity of 50–60 μmol photons m^−2^ s^−1^ and a 12-h light:12-h dark photoperiod. The normal culture medium (30‰ salinity) was aerated and refreshed every 2 days. For the high-salt stress (100‰) treatment, healthy thalli were placed in 500-mL conical flasks, with three thalli per flask (Chen et al., 2019). Samples were collected at specific time-points during the high-salt stress treatment (0 min, 15 min, 30 min, 2 h, and 4 h), after which samples were placed in normal seawater (R240) for a 4-h recovery period.

*Chlamydomonas reinhardtii* was grown in TAP medium and incubated with shaking at 100 rpm under the following conditions: light intensity, 50–60 μmol photons m^−2^ s^−1^; photoperiod, 14-h light:12-h dark; and temperature, 25°C. For the hypersaline stress treatment, samples were treated with 225 mM NaCl for 0, 15 min, 30 min, 2 h, 24 h, 48 h, and 72 h. The cell growth rate was calculated on the basis of the optical density at 750 nm (OD_750_) as previously described (Kumar et al., 2013).

### 2.2 Isolation of *PhNKA2* and vector construction

The complete *PhNKA2* cDNA sequence was cloned using specific primers (**Supplementary Table S1**). The amplified *PhNKA2* sequence was first inserted into the pMD19-T vector (TaKaRa, Dalian, China). The resulting recombinant plasmid and pChlamy_3 (Invitrogen, Carlsbad, USA) were digested with *Kpn*I and *Pst*I (TaKaRa, Japan) and then *PhNKA2* and the linearized pChlamy_3 vector were ligated to form the transformation vector (**Supplementary Figure S1**).

### 2.3 Yeast two-hybrid assays

Yeast two-hybrid assays were performed using the Clontech Yeast Two-Hybrid System (pGBKT7 and pGADT7) (Clontech, Palo Alto, CA, USA) as described by Wang et al. (2021). The *PhNKA2* open reading frame (ORF) was inserted into pGBKT7. The negative controls were the pGBKT7-Lam Control Vector and the pGADT7-T Control Vector. The positive controls were the pGBKT7-53 Control Vector and the pGADT7-T Control Vector. These controls were used for the self-activation and toxicity tests involving decoy genes. The positive clones (i.e., blue colonies) on the SD/−Trp/−Leu/X-α-Gal/AbA medium in plates were transferred to SD/−Trp/−Leu/−His/−Ade/X-α-Gal/AbA medium in plates to screen for protein interactions. The positive clones were selected for a one-to-one verification. The expression levels of the genes encoding interacting proteins were measured for the samples exposed to hypersaline stress (100‰).

### 2.4 *Chlamydomonas reinhardtii* transformation and screening of positive clones

The *PhNKA2* gene was inserted into *C. reinhardtii* using the glass bead transformation method (Kindle, 1990), after which the transformed *C. reinhardtii* cells were selected on medium containing hygromycin B (50 mg/mL) and propagated. Three randomly selected wild-type *C. reinhardtii* controls and three transgenic *C. reinhardtii* strains carrying *PhNKA2* were tested for paromomycin resistance. Genomic DNA was extracted from each sample for a PCR amplification using gene-specific primers to verify *PhNKA2* was present in the transgenic strains.

### 2.5 Transcriptional profiling of genes under hypersaline stress conditions

A qRT-PCR analysis was performed. The 20-μL reaction volume comprised the following components: 10 μL 2× SYBR Green Master Mix (Accurate Biotechnology (Hunan) Co., Ltd., Changsha, China), 0.4 μL forward and reverse primers (20 μmol L^−1^), 2 μL diluted template, 0.4 μL ROX Dye, and 6.8 μL ddH_2_O. The qRT-PCR conditions were as follows: 95°C for 30 s; 40 cycles of 95°C for 5 s and 54.3°C for 30 s. The fluorescence-based qRT-PCR analysis was completed using the ABI 7300 Real-Time PCR instrument (ABI, USA). The primers were designed according to the obtained gene sequences (**Supplementary Table S1**). In addition, to normalize gene expression levels, the housekeeping genes *Tubulin* (Wu et al., 2009) and *UBC* (Bing et al., 2014) were selected for *C. reinhardtii* and *P. haitanensis*, respectively.

### 2.6 Ion flux measurements

The Non-invasive Micro-test Technology (NMT) system (Younger USA LLC, Amherst, MA, USA) was used to measure the Na^+^ and K^+^ fluxes in the wild-type and transgenic *C. reinhardtii* cells before and after the salt stress treatment. The measurement solution was prepared as described by Chen et al. (2019). Ion-selective microelectrodes specific to Na^+^ and K^+^ were used to measure the ion fluxes. A 10-μL aliquot of healthy *C. reinhardtii* cells (OD_750_ approximately 0.3) was allowed to equilibrate in the equilibrium buffer for 1 min. The measurement duration was set to 10 min. The recorded values represent the average of each measurement at the same time-point for the same treatment.

### 2.7 Data processing and analysis

All treatments were performed using three biological replicates. The data were analyzed using the SPSS 22.0 software (SPSS Inc, USA). A one-way ANOVA and a *post hoc* LSD test were used to compare the differences between groups. The thresholds for significant and highly significant differences were *P* < 0.05 and *P* < 0.01, respectively.

## 3 Results

### 3.1 *PhNKA1* and *PhNKA2* expression in the gametophyte and sporophyte stages under hypersaline conditions

**Figure 1:**
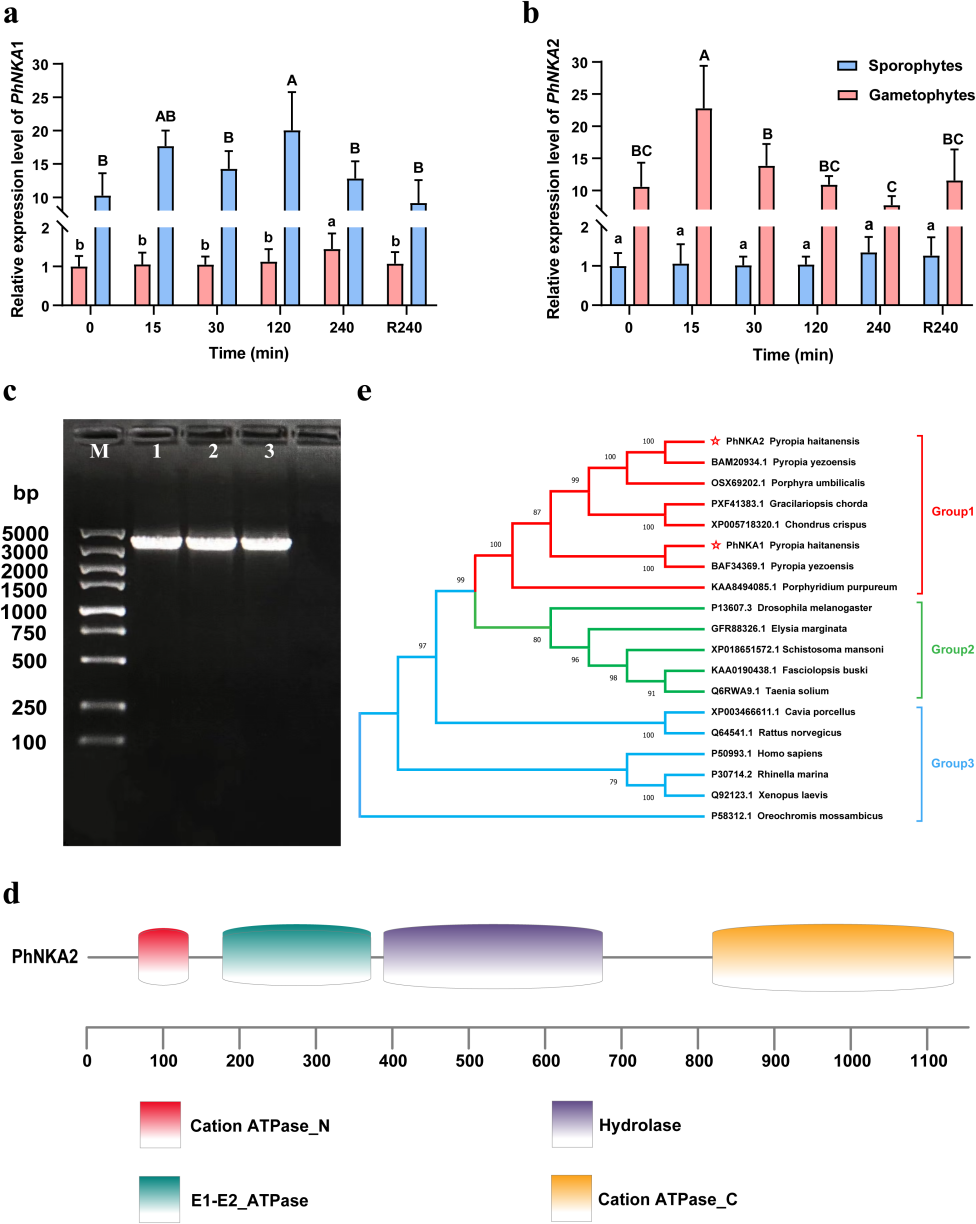
Characterization of the *PhNKA2* sequence and analysis of its expression in *Pyropia haitanensis*. Relative expression levels of **(a)** *PhNKA1* and **(b)** *PhNKA2* in the gametophyte and sporophyte stages under hypersaline conditions. Different letters indicate significant difference (p<0.05). The data for the thallus were obtained from one of our previous studies (Chen et al., 2023). **(c)** Agarose gel electrophoresis analysis of the *PhNKA2* PCR products; lanes 1–3 are three repeated samples. **(d)** Analysis of the *PhNKA2* domains. **(e)** Phylogenetic tree constructed using NKA amino acid sequences.

In response to the hypersaline stress treatment, *PhNKA1* was highly expressed in the sporophyte sporophytes stage, but it was expressed at lower levels in the gametophytes stage. The opposite expression pattern was also observed for *PhNKA2* (**Figures 1a, b**). The *PhNKA2* transcription level increased significantly in the *P. haitanensis* gametophyte thallus exposed to 100‰ hypersaline stress. The transcription of *PhNKA2* increased relatively early during the stress treatment, with a substantial increase of approximately 2.67-fold (relative to the control level) at 15 min after initiating the stress treatment. The transcription level tended to plateau, but remained high for the duration of the treatment period. Even after the 4-h recovery in normal seawater, the *PhNKA2* transcription level was significantly higher than the corresponding control level (**Figure 1b**).

### 3.2 Cloning and characterization of the *PhNKA2* sequence

On the basis of the results of the analysis of differential expression, we cloned the full-length *PhNKA2* gene sequence (**Figure 1c**). The *PhNKA2* ORF consists of 3,534 nucleotides, with the start codon ATG and the stop codon TAG. The encoded protein (1,177 amino acids) contains the following four conserved domains: cation-transporting ATPase at the N-terminus, E1/E2 ATPase, hydrolase, and cation-transporting ATPase at the C-terminus (**Figure 1d**). The phylogenetic analysis indicated that *PhNKA2* is highly homologous to sequences in other red algal species, including *P. yezoensis*, *P. umbilicalis*, *Chondrus crispus*, and *Gracilariopsis chorda* (**Figure 1e**). These findings suggest that *PhNKA2* is an evolutionarily conserved gene in red algae, especially among *Pyropia* species.

### 3.3 Screening and validation of proteins that interact with *PhNKA2* during the response of *P. haitanensis* thalli to hypersaline stress

**Figure 2:**
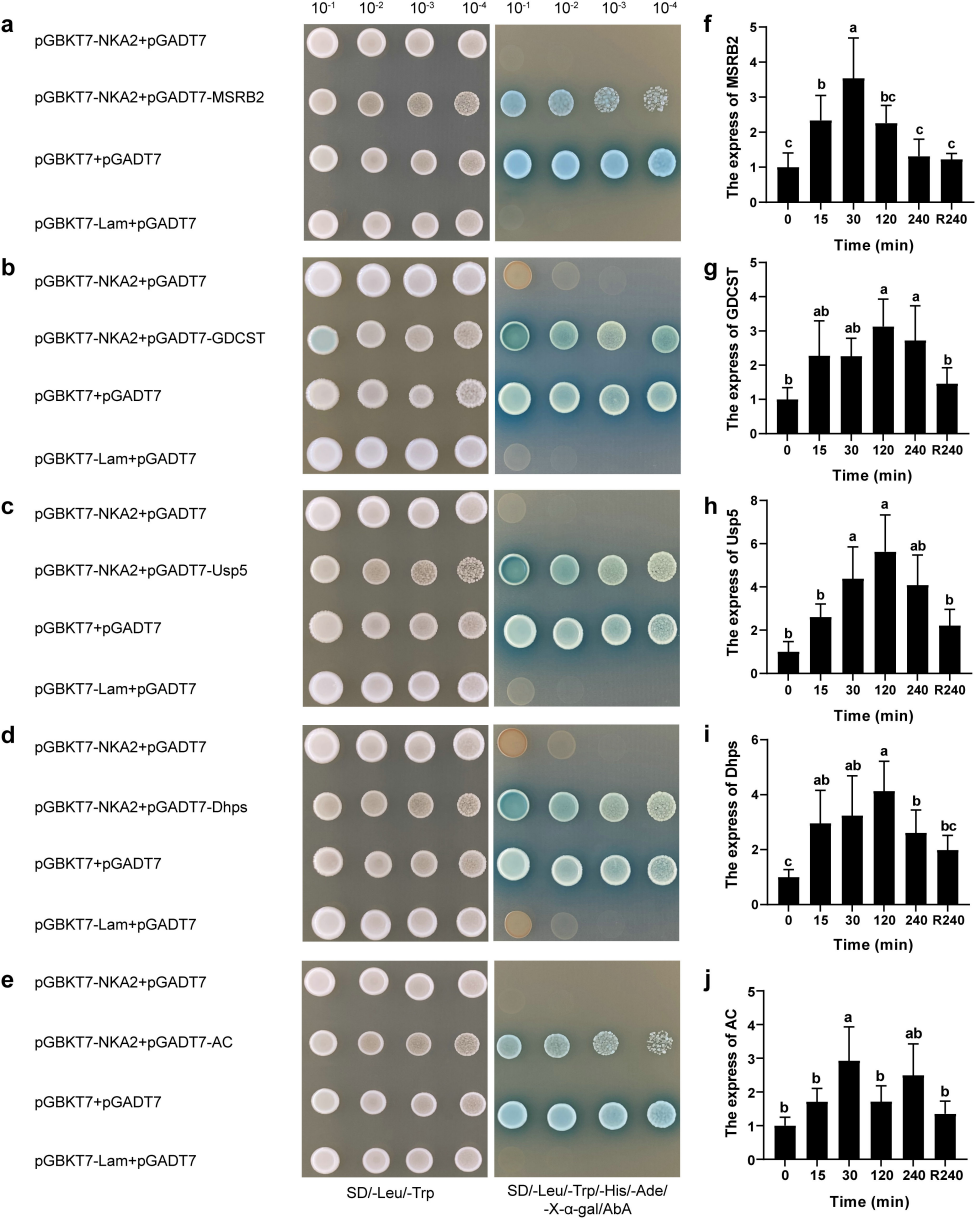
Identification of proteins that interact with *PhNKA2* and analysis of the differential expression of the corresponding genes. **(a)** Methionine sulfoxide reductase B2 (MSRB2), **(b)** glycine decarboxylase T protein (GDCST), **(c)** ubiquitin-specific protease (Usp5), **(d)** dihydroxyacetone phosphate synthase (Dhps), and **(e)** actin (AC) were identified as the proteins that can interact with *PhNKA2*. Relative transcript levels of the genes encoding **(f)** MSRB2, **(g)** GDCST, **(h)** Usp5, **(i)** Dhps, and **(j)** AC in *Pyropia haitanensis* thalli under hypersaline conditions were determined; pGBKT7 + pGADT7 were used as the positive control, whereas pGBKT7-Lam + pGADT7 were used the negative control. R240: 4-h recovery from salt stress in normal seawater. Different letters indicate significant differences among treatments (*P <* 0.05).

Following a one-to-one yeast two-hybrid verification using pGBKT7-NKA2, 17 positive clones were obtained (a total of 15 positive genes). After aligning the generated sequences, five genes encoding interacting proteins with annotation and complete ORF were identified, including genes for a ubiquitin-specific protease (Usp5), methionine sulfoxide reductase B2 (MSRB2), glycine decarboxylase T protein (GDCST), dihydroxyacetone phosphate synthase (Dhps), and actin (AC). The one-to-one spot plate verification confirmed that these five proteins can interact with *PhNKA2* (**Figures 2a–e**). The transcription of the genes encoding Usp5, MSRB2, GDCST, Dhps, and AC increased in the gametophytes thalli exposed to hypersaline stress (**Figures 2f–j**).

### 3.4 Functional verification of *PhNKA2* through heterologous expression

**Figure 3:**
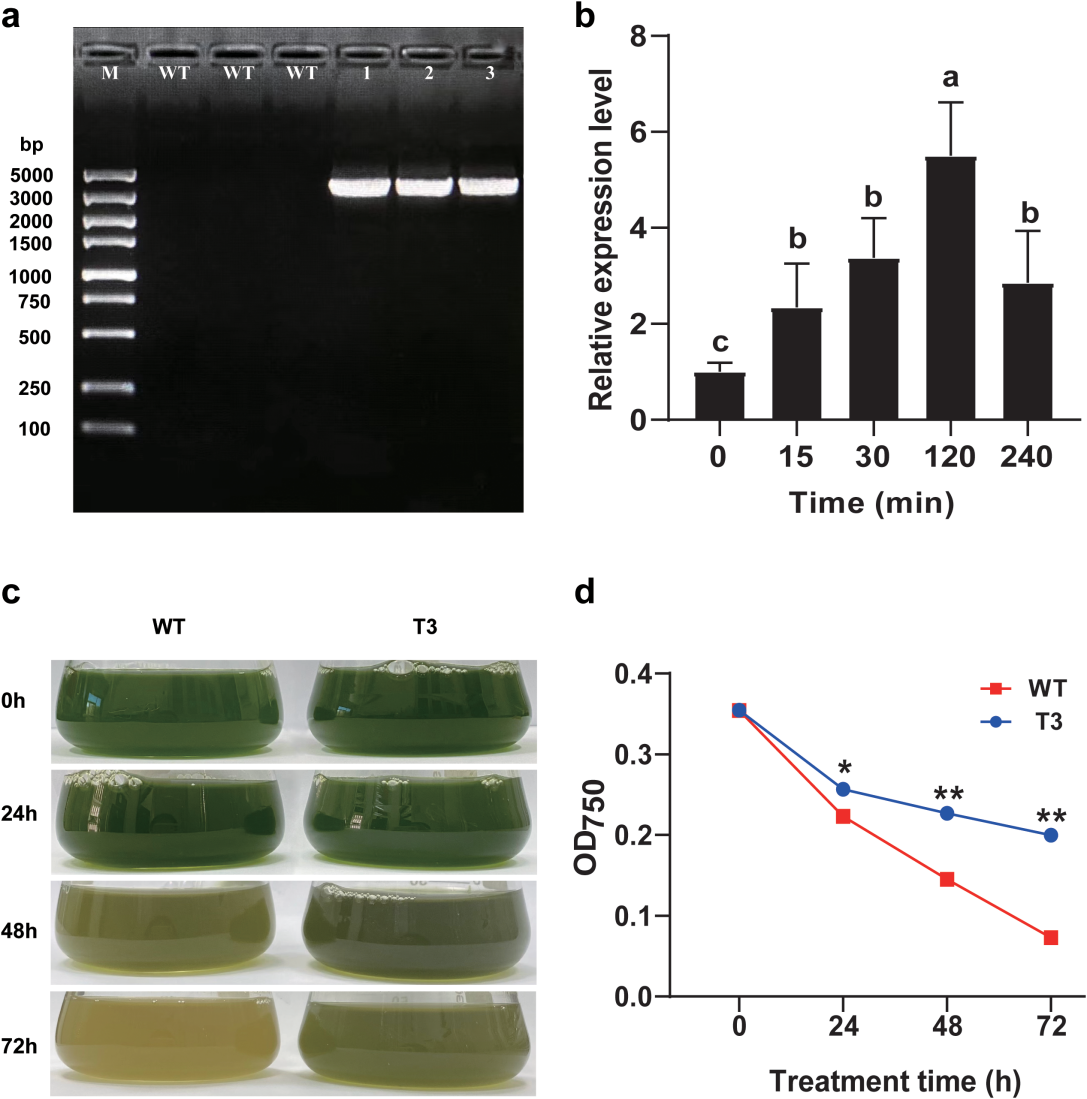
Verification of the transformation of *Chlamydomonas reinhardtii* with *PhNKA2*. **(a)** Agarose gel electrophoresis verification of the presence of *PhNKA2* in *C. reinhardtii*. **(b)** *PhNKA2* expression pattern in transgenic *C. reinhardtii* under hypersaline conditions. **(c)** Changes in the *C. reinhardtii* biomass in response to hypersaline stress. **(d)** Changes in the *C. reinhardtii* cell concentration in response to hypersaline stress. WT: wild-type *C. reinhardtii*; 1–3: transgenic *C. reinhardtii*. Each group comprised three replicates. Different lowercase letters indicate significant differences among treatments (*P* < 0.05). At each time-point, * and ** indicate significant differences between T3 and WT at the *P* < 0.05 and *P* < 0.01 levels, respectively.

The electrophoresis results confirmed that all of the candidate transgenic *C. reinhardtii* carried *PhNKA2*, while the wild-type *C. reinhardtii* strains did not (**Figure 3a**). Thus, the transgenic *C. reinhardtii* strains were successfully transformed with *PhNKA2*. The third sample (T3) was selected for further analysis. In the early stage of the salt stress treatment (15 min), the *PhNKA2* expression level increased significantly in the transgenic algae (*P* < 0.05). It peaked at 120 min, which was approximately 5.5-times higher than the control level. At the 240 min time-point, the *PhNKA2* expression level decreased slightly, but it was still significantly higher than the control level (approximately 2.9-times higher; *P* < 0.05) (**Figure 3b**).

To analyze the salt tolerance of the transgenic *C. reinhardtii* expressing *PhNKA2*, both wild-type and transgenic algae were treated with 225 mM NaCl for 72 h. The algal growth status was observed and the OD_750_ value was recorded. The hypersaline treatment significantly decreased the biomass of the wild-type and transgenic *C. reinhardtii* strains. However, the biomass was consistently lower for the wild-type algae than for the transgenic algae. Moreover, as the duration of the salt stress treatment increased, the difference in the biomass between the two groups increased. Accordingly, the overexpression of *PhNKA2* enhanced the salt tolerance of the transgenic *C. reinhardtii* strains (relative to the salt tolerance of the wild-type controls) (**Figures 3c, d**).

**Figure 4:**
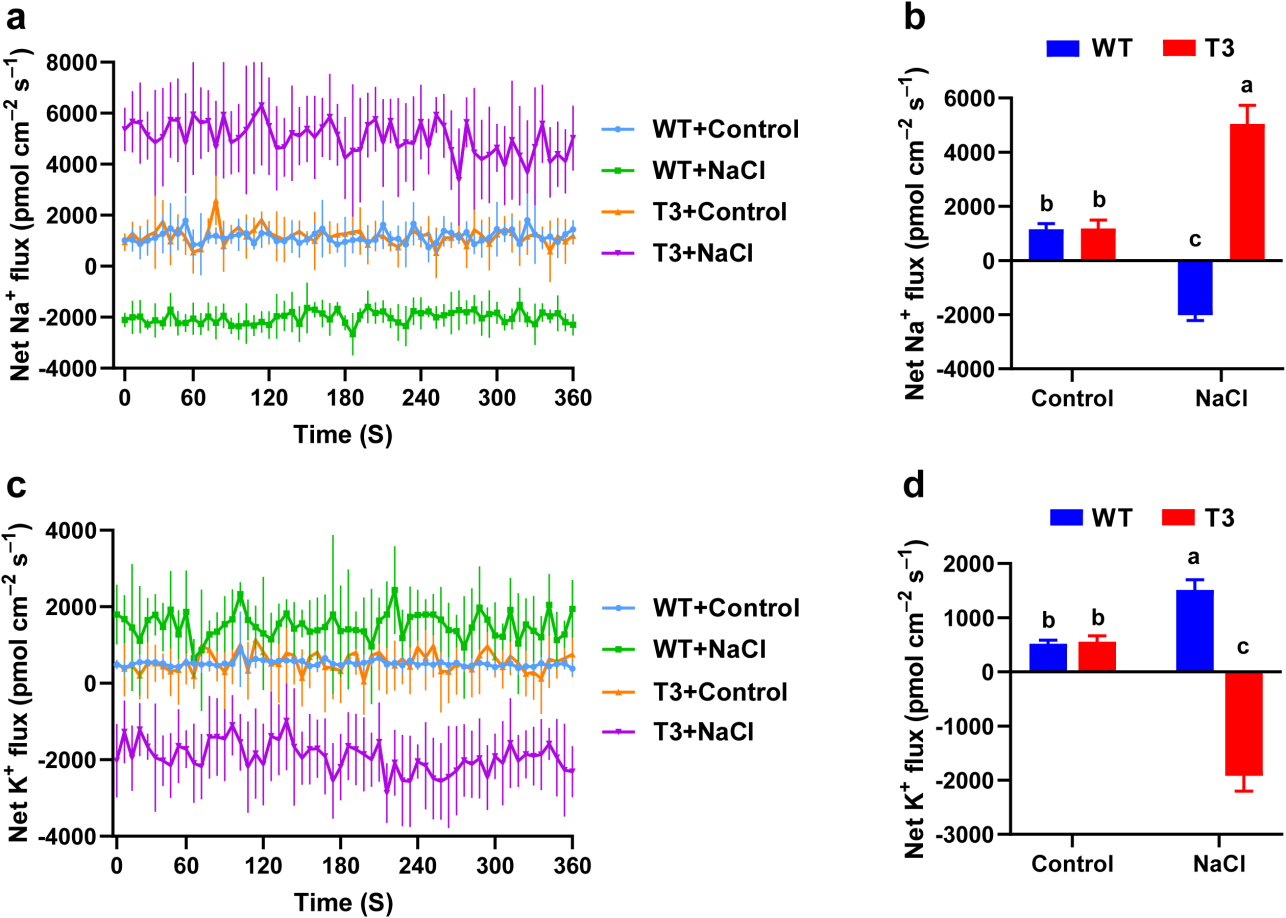
Effects of hypersaline stress on the net Na^+^ and K^+^ fluxes in *C. reinhardtii*. **(a)** Real-time dynamic transport of Na^+^. **(b)** Mean Na^+^ flux rate. **(c)** Real-time dynamic transport of K^+^. **(d)** Mean K^+^ flux rate. WT: wild-type *C. reinhardtii*; T3: transgenic *C. reinhardtii*; Control: normal physiological conditions; NaCl: hypersaline stress (225 mM). Different lowercase letters indicate significant differences (*P <* 0.05).

The changes in the Na^+^ and K^+^ fluxes in the *PhNKA2*-overexpressing *C. reinhardtii* strains exposed to salt stress were measured using the NMT system. Under normal culture conditions, Na^+^ efflux was detected in the wild-type and transgenic *C. reinhardtii* samples, with average efflux rates of 1,155.36 and 1,182.38 pmol cm^−2^ s^−1^, respectively. The 15-min hypersaline stress (225 mM) treatment induced a significant influx of Na^+^ in the wild-type *C. reinhardtii* cells, with an average influx rate of −2,017.01 pmol cm^−2^ s^−1^ (**Figures 4a, b**). In contrast, the hypersaline stress treatment resulted in a significant efflux of Na^+^ from the transgenic *C. reinhardtii* cells, with an average efflux rate of 5,040.49 pmol cm^−2^ s^−1^.

Furthermore, under normal conditions, K^+^ efflux was observed in the wild-type and transgenic *C. reinhardtii* cells, with average efflux rates of 517.54 and 558.74 pmol cm^−2^ s^−1^, respectively. The exposure to hypersaline stress led to the significant efflux of K^+^ from the wild-type *C. reinhardtii*, with an average efflux rate of 1,511.21 pmol cm^−2^ s^−1^, which was in contrast to the significant influx of K^+^ detected for the transgenic *C. reinhardtii* cells, with an average influx rate of −1,919.13 pmol cm^−2^ s^−1^ (**Figures 4c, d**). Hence, the overexpression of *PhNKA2* in *C. reinhardtii* altered the Na^+^ and K^+^ fluxes, especially under saline conditions. Compared with the wild-type controls, the transgenic algae were better able to maintain the K^+^/Na^+^ homeostasis, which may have contributed to their enhanced salt tolerance.

## 4 Discussion

### 4.1 Structural and functional analysis of *PhNKA2* sequences

The enzyme Na^+^/K^+^-ATPase, which belongs to the P-type cation-transporting ATPase family, undergoes significant conformational changes while maintaining the K^+^/Na^+^ balance (Morth et al., 2011). The identified Na^+^/K^+^-ATPases in animals, red algae, and green algae consist of four structural domains (E1/E2 ATPase, hydrolase, and N-terminal and C-terminal cation-transporting ATPases) (Barrero-Gil et al., 2005; Kumari and Rathore, 2020; Li et al., 2023). These domains are essential for the ion transport and ATP hydrolysis activities of Na^+^/K^+^-ATPase. Such as, the α subunit of Na^+^/K^+^-ATPase alternates between the E1/E1P and E2P/E2 states, thereby playing a major role in the translocation of cytoplasmic Na^+^ and extracellular K^+^. During the conformational rearrangement from E2 to E1, the β subunit helps stabilize the α subunit (Dempski et al., 2006). The cloning of *PhNKA2* revealed it also encodes the four conserved domains characteristic of Na^+^/K^+^-ATPases (**Figure 1d**). Additionally, *PhNKA2* possesses α-helices and β-sheets, indicating that it likely has a catalytically active α subunit and a stabilizing β subunit. Thus, *PhNKA2* may be functionally similar to the Na^+^/K^+^-ATPases in other organisms, in which the α subunit catalyzes the transport of ions and the β subunit promotes enzyme stability.

### 4.2 Analysis of the differential expression of *PhNKA1* and *PhNKA2* under hypersaline stress conditions

The differential expression analysis showed that following the high-salt stress (100‰) treatment, the *PhNKA2* expression level in *P. haitanensis* thalli started to significantly increase at 15 min, but then gradually decreased, while still remaining higher than the control level (**Figure 1b**). Moreover, the Na^+^/K^+^-ATPase activity in *P. haitanensis* increased significantly soon after the salt stress treatment was started (i.e., 15 min) and was maintained at a high level at the subsequent time-points. However, the inhibition of the Na^+^/K^+^-ATPase activity reportedly results in a significant accumulation of Na^+^ and a sharp decrease in the K^+^ content in the thalli (Chen et al., 2022). Furthermore, transgenic *C. reinhardtii* strains carrying *PhNKA2* had higher survival rates than the wild-type strains under hypersaline conditions (**Figure 3**). Additionally, there was a significant Na^+^ efflux and a significant K^+^ influx in the transgenic strains, whereas the opposite trends were detected in the wild-type *C. reinhardtii* strains (**Figure 4**). These findings imply that Na^+^/K^+^-ATPase plays a crucial role in the response of algae to salinity. Additionally, CRISPR/Cas9-based precise genome editing technology is a highly efficient and precise genetic engineering technique that has rapidly developed over the past decade (Gao, 2021). This technology is widely used for gene function and trait analysis in economically important crops. Therefore, in the future, a DNA vector-based CRISPR/Cas precise genome editing system for *P. haitanensis* should be established as soon as possible to verify the biological functions and differentiation mechanisms of different Na^+^/K^+^-ATPase.

Interestingly, *PhNKA1* and *PhNKA2* had the opposite expression patterns in *P. haitanensis*. Specifically, *PhNKA1* was expressed at high and low levels in the sporophytes stage and the gametophyte stage, respectively. Uji et al. (2012) observed a similar phenomenon in *P. yezoensis.* This difference in expression patterns may be because the salt stress level is relatively low in the sporophytes stage; the higher salt stress level during the thallus stage necessitates the increased production of *PhNKA2* to maintain the ionic balance. However, unlike the expression patterns in response to salt stress, the expression levels of both *PyKPA1* and *PyKPA2* increase in the gametophytes of cold-stressed *P. yezoensis*. This suggests that different Na^+^/K^+^-ATPases are regulated by distinct mechanisms during responses to different abiotic stresses. The specific mechanisms underlying these differences warrant further investigation.

### 4.3 Functional analysis of *PhNKA2*-interacting proteins

In addition to pumping Na^+^ and K^+^ across the cell membrane, Na^+^/K^+^-ATPase is also involved in the assembly of various protein complexes on the plasma membrane, which affects cell growth, gene expression, and signal transduction (Wen and Wan, 2021; Sun et al., 2023). In the current study, *PhNKA2* was revealed to interact with several proteins, namely Usp5, MSRB2, GDCST, Dhps, and actin. Furthermore, the expression of the genes encoding these proteins was rapidly induced by salt stress (**Figure 2**). Deubiquitination-related enzymes, such as Usp5, remove ubiquitin from ubiquitinated proteins, thereby regulating various cellular processes (Komander et al., 2009). Zhou et al. (2012) reported that the *Arabidopsis ubp16* mutant is sensitive to salinity at the seedling and adult stages, with a higher Na^+^ content in the mutant leaves than in the wild-type leaves. Further analyses indicated that ubp16 stabilizes SERINE HYDROXYMETHYLTRANSFERASE1, which is a key protein for the salt tolerance of Arabidopsis, by removing the conjugated ubiquitin. Sun et al. (2023) recently determined that the α1 subunit of Na^+^/K^+^-ATPase may form a complex with ubiquitin-specific peptidase 22 (USP22) on the membrane, which is conducive to the subsequent deubiquitination and stabilization of sirtuin 1 and leads to the activation of downstream autophagy-related signaling. Deubiquitinases or ubiquitin ligases do not appear to regulate the degradation of Na^+^/K^+^-ATPase, but they can serve as carriers for ubiquitin-dependent intracellular transport (Wen and Wan, 2021). Therefore, the interaction of the deubiquitinating enzyme USP5 with *PhNKA2* might be critical for the deubiquitination and stabilization of key proteins in *P. haitanensis* growing in saline environments.

Salinity stress can induce the excessive accumulation of reactive oxygen species (ROS) in *Pyropia* cells (Wang et al., 2019). Therefore, the timely and efficient scavenging of ROS is crucial for maintaining the normal function of proteins mediating the response to salt stress. Methionine (Met) is highly prone to oxidation, which results in the formation of methionine sulfoxide (MetSO) and changes to protein activity or even loss of activity (Luo and Levine, 2009). The methionine sulfoxide reductase (MSR) family includes MSRB, which specifically catalyzes the reduction of the R-type MetSO and helps to maintain the MetSO content at an appropriate level to limit the oxidative damage to proteins due to excessive MetSO (Tarrago et al., 2009). The 24 proteins that can interact with *Arabidopsis* MSRB1 have surface-exposed Met residues and a higher content than average Met content, implying they are highly susceptible to ROS-related oxidation and dependent on MSRB for repairs (Tarrago et al., 2011). For example, because of the presence of Met-rich domains, heat shock proteins (HSPs) were among the first plant proteins predicted to be substrates of MSR, which helps maintain HSP structures and functions by reducing MetSO in HSPs (Gustavsson et al., 2002). Cui et al. (2022) reported that MsrB2 contributes to the drought tolerance of tomato by interacting with catalase 2 to promote the scavenging of ROS (Cui et al., 2022). Additionally, the overexpression of *AtDHPS* increases the *Arabidopsis* seed germination rate under oxidative stress conditions (Navarrete et al., 2012). The photorespiration-related GDC complex, which also facilitates an efficient redox transfer, is important for plant salt tolerance (Igamberdiev et al., 2001; Abogadallah, 2011). Accordingly, the interaction of *PhNKA2* with MSRB2, DHPS, or GDCST might prevent the oxidation of *PhNKA2*, which mediates the salt tolerance of *P. haitanensis* thalli.

The actin-based cytoskeleton also interacts with Na^+^-K^+^-ATPases (Morrow et al., 1989; Ferrandi et al., 1999; Gomes and Soares-Da-Silva, 2002). The cytoskeleton–Na^+^-K^+^-ATPase interaction is regulated by ankyrin, a cytoskeletal protein (Nelson and Veshnock, 1987). The dopamine-induced inhibition of the Na^+^-K^+^-ATPase activity depends on the interaction between the Na^+^-K^+^-ATPase and the actin cytoskeleton (Gomes and Soares-Da-Silva, 2002). Actin depolymerization can also stimulate membrane trafficking involving Na^+^-K^+^-ATPase (Namekata et al., 2008). The actin-based cytoskeleton is an essential, dynamic component of eukaryotic cells, with a vital role related to the abiotic stress tolerance of intertidal seaweed species *Pyropia* (Shi et al., 2019; Kong et al., 2021). The abundance of three actin and tubulin proteins in *P. haitanensis* increases at moderate desiccation levels (Shi et al., 2019). These results suggested a possible relationship between actin and *PhNKA2* functions in *P. haitanensis* in saline environments. These potential relationships and the associated mechanisms in *Pyropia* species will need to be characterized in future studies.

## 5 Conclusion

In this study, the gene encoding the full-length sequence of an animal-type Na^+^-K^+^-ATPase (*PhNKA2*) was cloned from *P. haitanensis*, which encodes four typical domains. It’s expression level were significantly induced by hypersaline stress. The overexpression of *PhNKA2* in transgenic *C. reinhardtii* significantly enhanced the efflux of Na^+^ and the influx of K^+^ under saline conditions, ultimately leading to increased salt tolerance. Further analyses revealed that in addition to maintaining the K^+^/Na^+^ balance, *PhNKA2* may also stabilize important salt tolerance-related proteins through interactions with deubiquitinases. Furthermore, its interactions with MSRB2, DHPS, or GDCST provide protection from oxidation, while its interactions with actin influence trans-membrane transport.

## Data Availability

The original contributions presented in the study are included in the article/**Supplementary Material**. Further inquiries can be directed to the corresponding authors.
