# Supplementary material for: An animal-type Na+/K+-ATPase, PhNKA2, is involved in the salt tolerance of the intertidal macroalga Pyropia haitanensis
Source: Front Plant Sci. 2025 Apr 28;16:1571241. doi: 10.3389/fpls.2025.1571241 (PMC12066774; doi:10.3389/fpls.2025.1571241)
Supplement: Supplementary file 1 [file DataSheet1.zip › Supplementary/Supplementary Table S1.docx]

**Supplementary Table S1 Specific primers used in the present experiments**

| Species | Specific primers | Sequence of primers |
| --- | --- | --- |
| *Pyropia haitanensis* | *PhNKA2F* | ATGGCGCCCCCGGCGGCC |
|  | *PhNKA2R* | CTACCAGTACGTGTTGTACCGGAG |
|  | *PhNKA2QF* | TCCTCTGCTTTATCGGCTT |
|  | *PhNKA2QR* | AACTTTTCCATCGTCTTCTCG |
|  | *PhMSRB2QF* | AGTATCGCATCCTCCGTCAAAA |
|  | *PhMSRB2QR* | TCATCCCAAACGAGAAGTCCG |
|  | *PhGDCSTQF* | GGGAGGACGGCTTTGAGATT |
|  | *PhGDCSTQR* | CAGCTGCTTCATGATGACGGT |
|  | *PhACQF* | GCTGTTCAAGCCCGACCTC |
|  | *PhACQR* | GCCACCAATCCACACCGA |
|  | *PhDhpsQF* | ACCATCTCAGACCATCCGCACG |
|  | *PhDhpsQR* | TCCGACTTGACCAGCACCGC |
|  | *PhUsp5QF* | TCGGTGGAGATGCTGACTGCG |
|  | *PhUsp5QR* | AGGTCGTCCGCGTGGCTAAA |
|  | *PhUBCF* | TCACAACGAGGATTTACCACC |
|  | *PhUBCR* | GAGGAGCACCTTGGAAACG |
| *Chlamydomonas reinhardtii* | *TubulinQF* | CTCGCTTCGCTTTGACGGTG |
|  | *TubulinQR* | CGTGGTACGCCTTCTCGGC |
