# Supplementary figures and images for: An animal-type Na+/K+-ATPase, PhNKA2, is involved in the salt tolerance of the intertidal macroalga Pyropia haitanensis
Source: Front Plant Sci. 2025 Apr 28;16:1571241. doi: 10.3389/fpls.2025.1571241 (PMC12066774; doi:10.3389/fpls.2025.1571241)

**Supplementary Figure S1** *Chlamydomonas reinhardtii* vector containing *PhNKA2*

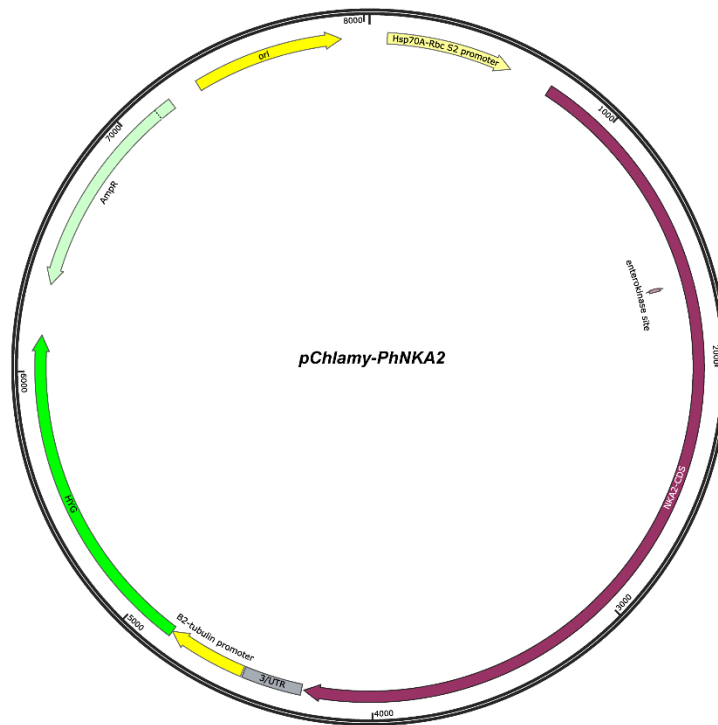

Supplement: Supplementary file 1 [file DataSheet1.zip › Supplementary/Supplementary Figure S1 Chlamydomonas reinhardtii vector containing PhNKA2.pdf]
